# Draft Genome Sequence of a Filamentous Anoxygenic Phototrophic Bacterium, “*Candidatus* Roseilinea sp. Strain NK_OTU-006,” Recovered from Metagenomic Data of a Hot Spring Microbial Mat

**DOI:** 10.1128/MRA.01104-20

**Published:** 2020-12-10

**Authors:** Joval N. Martinez, Shigeru Kawai, Mohit Kumar Saini, Marcus Tank, Satoshi Hanada, Vera Thiel

**Affiliations:** a Department of Biological Sciences, Graduate School of Science, Tokyo Metropolitan University, Hachioji, Tokyo, Japan; b Department of Natural Sciences, College of Arts and Sciences, University of St. La Salle, Bacolod City, Negros Occidental, Philippines; c Institute for Extra-Cutting-Edge Science and Technology Avant-Garde Research (X-star), Japan Agency for Marine-Earth Science & Technology (JAMSTEC), Yokosuka, Kanagawa, Japan; d Leibniz-Institut DSMZ-Deutsche Sammlung von Mikroorganismen und Zellkulturen GmbH, Braunschweig, Germany; University of Maryland School of Medicine

## Abstract

We report here the metagenome-assembled draft genome of an uncultured filamentous anoxygenic phototroph of the phylum *Chloroflexota* (formerly *Chloroflexi*), “*Candidatus* Roseilinea sp. strain NK_OTU-006,” recovered from hot spring-associated microbial mats. The 3.6-Mb genome is estimated to be 94% complete and comprises 117 contigs encoding 3,203 predicted genes, including a full-length rRNA operon.

## ANNOUNCEMENT

All known culturable filamentous anoxygenic phototrophic (FAP) bacteria within the phylum *Chloroflexota* are affiliated with the class *Chloroflexia.* However, metagenomic studies of hot spring-associated microbial mats discovered a novel FAP outside this class (1, 2). This still-uncultured organism was first described as an *Anaerolinea*-like bacterium and was later tentatively named “*Candidatus* Roseilinea gracile” (3). A recent evolutionary study of a related uncultured representative, the metagenome-assembled genome (MAG) JP3_7, proposed that both phototrophs from geographically different hot springs belong to the same novel “*Candidatus*” class, “*Candidatus* Thermofonsia,” of which no validly described species have been reported so far (4). Here, we report the draft genome of a third hot spring-associated MAG representative of this novel class, “*Candidatus* Roseilinea sp. strain NK_OTU-006.”

Phototrophic microbial mats were collected from Nakabusa hot springs in Nagano Prefecture, Japan (36°23′33.1″N, 137°44′53.0″E), at an overlying water temperature of ∼56°C. Genomic DNA was isolated from the mats using a PowerBiofilm DNA extraction kit (Qiagen, Inc.). Sequencing libraries were prepared using a KAPA Hyperplus library prep kit (Kapa Biosystems, Inc.) and were sequenced via an Illumina MiSeq instrument (2 × 300 bp) at FASMAC (Japan). Raw metagenome sequences (32,921,964 reads) were quality filtered using Sickle v.1.33 (5), trimmed using FASTX-Toolkit v.0.0.13.2 (6), and assembled using SPAdes v.3.7.1 (7) with default parameters. Tetranucleotide binning of assembled contigs was employed using Emergent Self-Organizing Map (ESOM) as described previously (8). Bin identity and completeness were assessed based on the presence of phylogenetic marker genes and taxonomic affiliations implemented in AmphoraNet (9) and CheckM (10).

The 3.6-Mb draft genome sequence consists of 63% G+C content and is estimated to be 94.1% complete, as determined by CheckM (10). Genome annotation by Prokka v.1.2 (11) comprises 117 contigs, with 3,203 predicted genes, 3,061 protein-coding sequences, 44 tRNAs, and 3 rRNAs (5S, 16S, 23S). The full-length 16S rRNA gene sequence from the NK_OTU-006 bin shows the highest identity (95%) with “*Ca*. Roseilinea gracile” (3). However, the average nucleotide identity (ANI) values show 78% and 99% similarities to “*Ca.* Roseilinea gracile” and JP3_7 MAGs, respectively. NK_OTU-006 also shows low ANI (≤78%) and 16S rRNA gene sequence identity (≤87%) to any described isolates from the classes *Anaerolinea* (12, 13) and *Chloroflexia* (14, 15).

MAG NK_OTU-006 contains genes encoding a type 2 photosynthetic reaction center (*pufLMC*) and light-harvesting complex 1 (*pufAB*), which is consistent with genes found in “*Ca.* Roseilinea gracile” (3) and JP3_7 MAGs (4). Similar to the red filamentous anoxygenic phototroph *Roseiflexus* spp. (14, 16), no genes encoding chlorosomes were recovered, while genes required for bacteriochlorophyll *a* and carotenoid biosynthesis were detected. Homologous genes encoding the major electron transport complexes for aerobic respiration and ACIII-like genes needed for phototrophy were also found. This finding is consistent with other those for phototrophic *Chloroflexia* (16–18).

Unlike many phototrophic *Chloroflexia* members, no genes for any known autotrophic carbon fixation pathways were detected, indicating a heterotrophic lifestyle (14–16). Genes for sulfide oxidation and nitrogen fixation were not recovered from the binned genome. However, uptake and bidirectional hydrogenase genes were found in the MAG, suggesting the ability to metabolize H_2_. The genomic information of “*Ca.* Roseilinea sp. NK_OTU-006” facilitates understanding of the lifestyle of the underrepresented lineage of phototrophic *Chloroflexota*. A metabolic model is accessible at https://kbase.us/n/59604/258/.

### Data availability.

This whole-genome shotgun project has been deposited at DDBJ/ENA/GenBank under the accession number NZ_JAAFGM000000000. The raw sequence reads are available in the Sequence Read Archive (SRA) under database accession number SRR12784215 and BioProject accession number PRJNA603154.
